# Calabrian Goji vs. Chinese Goji: A Comparative Study on Biological Properties

**DOI:** 10.3390/foods6040030

**Published:** 2017-04-10

**Authors:** Mariarosa Ruffo, Ortensia Ilaria Parisi, Fabio Amone, Rocco Malivindi, Domenico Gorgoglione, Filomena De Biasio, Luca Scrivano, Vincenzo Pezzi, Francesco Puoci

**Affiliations:** 1 Macrofarm s.r.l., c/o Department of Pharmacy, Health and Nutrition Sciences, University of Calabria, 87036 Rende (CS), Italy; mariarosaruffo8@gmail.com (M.R.); ortensiailaria.parisi@unical.it (O.I.P.); amonefabio@gmail.com (F.A.); rocco.malivindi@unical.it (R.M.); v.pezzi@unical.it (V.P.); 2 Department of Pharmacy, Health and Nutritional Sciences, University of Calabria, 87036 Rende (CS), Italy; luca.scrivano@unical.it; 3 Evra s.r.l., Località Galdo Zona Industriale Lotto 20, 85044 Lauria (PZ), Italy; info@evraitalia.it; 4 Osun Solutions s.r.l., Località Galdo, 85044 Lauria (PZ), Italy; lab@osunsolutions.it

**Keywords:** Calabrian Goji extract, DPPH, ABTS, ORAC, Nitric Oxide Radical Scavenging, cholinesterase inhibition, Zeaxanthin, antioxidant, anti-inflammatory

## Abstract

*Lycium barbarum* (Goji) fruits are mainly cultivated in northwestern China and are well known for their beneficial and healthy effects. In this work, the biological and functional properties of Calabrian Goji extract, obtained from Goji berries cultivated in the Sibari Plain (in the Italian region of Calabria), were demonstrated. In order to evaluate the use of this extract as a food supplement for cognitive and mental disorders, the quantification of Carotenoids as Zeaxanthin equivalents was made. The antioxidant activity was investigated by evaluating the scavenging properties against 2,2′-diphenyl-1-picrylhydrazyl (DPPH) and 2,2′-Azinobis-(3-ethylbenzothiazoline-6-sulfonic acid) (ABTS) radicals and by performing the ORAC (Oxygen Radical Absorbance Capacity) assay. The inhibition of lipid peroxidation was quantified by bleaching test and the ability to inhibit acetylcholinesterase enzyme and to scavenge nitric oxide radical was also evaluated. All the results were compared to those obtained from a Chinese Goji extract used as a reference. Based on the reported data, Calabrian Goji might be used as a food supplement with a possible application in cognitive disorders, mental impairments and other neurodegenerative diseases, due to its biological properties and the high levels of Carotenoids.

## 1. Introduction

*Lycium barbarum* fruits, also known as Goji berries, are widely used in traditional Chinese medicine, as an herbal remedy, and in minor pathological conditions. The nutritive and antioxidant activities of this fruit were already known in ancient times. The recent diffusion of Goji on the Italian functional food market owes its success to the many beneficial effects attributed to the product, such as antioxidant, immunomodulatory and neuroprotective properties [1]. A wide range of bioactive molecules, like Phenolic compounds, Vitamin C, and Carotenoids are largely present in the fruit, as well as in Goji leaves [2]. Among all, Carotenoids contribute from 0.03% up to 0.5% of the dry fruit weight, being responsible for its red-orange colour [3]. The class of Carotenoids include β-Carotene, Neoxanthin, Cryptoxanthin that are present in lower concentrations in Goji berries compared to Zeaxanthin, which is the most represented Carotenoid in the fruit, ranging between 31% and 56% of the total Carotenoid content, in the form of Dipalmitin Zeaxanthin [4]. Zeaxanthin is a xanthophyll pigment that can be found in egg yolk, as well as coloured fruits and vegetables. This Carotenoid, together with Lutein and Cryptoxanthin, accumulates primarily in the frontal and occipital cortices of the brain, where it exerts a neuroprotective activity, and lower concentrations are associated with lower cognitive functions [5]. Since Zeaxanthin, as its isomer (Lutein), passes the blood-retina barrier of the eye, it can also accumulate in the macula in higher concentrations than in other tissues, and it can prevent macular degeneration, acting as a filter of the blue light that enters the eye [6]. For this reason, they are also known as macular pigments. Many studies report a close correlation between serum levels of Zeaxanthin and its concentration in the macula. Moreover, the optical density of the macular pigment is strictly related to the amount of Carotenoid located in the brain. Thus, it can be a tool for Zeaxanthin and Lutein brain concentration determination [7]. This evidence is in line with age-related retinal degeneration in subject with cognitive impairment conditions [8]. Although the molecular processes involved in Zeaxanthin neuroprotection are not well-known, the proposed mechanisms include antioxidant activity and scavenging activity against free radical and reactive oxygen species, reduction of the oxidative stress, regulation of the inflammatory pathways and modulation of the synaptic membrane stability [9]. Indeed, it has been largely assessed that oxidative stress and inflammation are the secondary cause of Alzheimer’s disease, and of other conditions related to memory loss and cognitive impairments [10]. Primary hallmarks of the disease are the accumulated extracellular amyloid beta and intraneuronal phosphorylated tau protein. All these evidences suggested that dietary integration with products containing antioxidant and anti-inflammatory agents, such as Zeaxanthin, may prevent the oxidative stress and the inflammatory damage of neural tissues and thus may be of benefit in different cognitive disorders.

The purpose of this research was to study the beneficial properties of Goji fruit cultivated in the Sibari Plain (in the Italian region of Calabria) and investigate the chemical composition of this fruit grown in a different climatic area, such as the Mediterranean region. Two types of Goji berries extract were compared: one obtained from Goji fruit cultivated in the Sibari Plain and the other one obtained from Chinese Goji berries. The amount of Carotenoids was quantified and expressed as Zeaxanthin equivalents, and the antioxidant and anti-inflammatory activities of Calabrian Goji extract were also investigated. All of the experiments were conducted in order to analyze this extract as a food supplement that could be used for the treatment of cognitive and mental disorders.

## 2. Materials and Methods

### 2.1. Materials

2,2′-Diphenyl-1-picrylhydrazyl radical (DPPH), 2,2′-Azinobis-(3-ethylbenzothiazoline-6-sulfonic acid) (ABTS), Potassium persulfate, Dipotassium hydrogen phosphate, Potassium dihydrogen phosphate, (±)-6-Hydroxy-2,5,7,8-tetramethylchromane-2-carboxylic acid (Trolox), Fluorescein, 2,2′-azobis-(2-amidinopropane) dihydrochloride (AAPH), β-Carotene, Linoleic acid, Tween 20, Acetylthiocholine iodide (ATChI), 5,5′-Dithio-bis(2-nitrobenzoic-acid) (DTNB), Torpedo californica (electric eel) Acetylcholinesterase (Type-VI-S, EC 3.1.1.7 (AChE)), Sulfanilamide, *N*-(1-naphthyl) ethylenediamine dihydrochloride, Sodium nitroprusside, Phosphoric acid (H_3_PO_4_), Zeaxanthin (Z) and Sodium chloride (NaCl) were obtained from Sigma-Aldrich (Sigma Chemical Co., St Louis, MO, USA).

All the solvents were high-performance liquid chromatography (HPLC)-grade and provided by Sigma-Aldrich (Sigma Chemical Co., St. Louis, MO, USA).

Calabrian Goji fruits were obtained from Biocal s.r.l. (Calabria, Italy), while Chinese Goji fruits, cultivated in China, were imported and provided by Biocal s.r.l. Dry extracts of both fresh fruits were prepared by Evra s.r.l., following an extraction procedure covered by professional secrecy. The extracts have been lyophilized to dry powder before proceeding with biological properties evaluation. In each experiment the Chinese Goji extract powder was used as a reference.

### 2.2. Instrumentation

UV-Vis absorption spectra were obtained with a Jasco V-530 UV/Vis spectrometer (Jasco, Lecco, Italy).

ORAC assay was carried out using a Synergy H1 Hybrid Reader; Bio-Tek Instruments, Inc., Winooski, VT, USA.

### 2.3. Scavenging of DPPH Free Radical

The DPPH free radical scavenging assay was carried out as described in literature with slight modifications [11]. In the adopted protocol, 1 mL of an aqueous solution 1.5 mg/mL of each tested Goji extract was mixed with 4 mL of an ethanolic DPPH solution (200 μM) and, then, 6 mL of ethanol were added. A control was prepared in the same experimental conditions using 1 mL of distilled water. All the samples were incubated in the dark and, after 15 min, the absorbance related to the residual DPPH concentration was measured at 517 nm.

The radical scavenging activity of the tested Goji extracts was expressed as inhibition percent, which was calculated according to the following Equation (1):

(1) $$Inhibition\;\left(\%\right)=\left(\frac{A_{0}-A_{1}}{A_{0}}\right)\times 100\;$$

where *A*_0_ is the absorbance of the control and *A*_1_ is the absorbance of the samples.

All determinations were carried out in triplicate and data were expressed as standard error of the mean (SEM).

### 2.4. ABTS Radical Scavenging Assay

2,2′-Azinobis-(3-ethylbenzothiazoline-6-sulfonic acid) (ABTS) radical scavenging activity was investigated by absorbance measurement of the radical cation (ABTS^•+^) according to literature with some modifications [12]. This method is based on the ability of antioxidants to quench the blue-green ABTS radical cation, a chromophore with a characteristic absorption at 734 nm.

ABTS^•+^ was generated by the reaction of an aqueous ABTS solution (7 mM) with 2.45 mM Potassium persulfate (K_2_S_2_O_8_). The obtained mixture was left at room temperature and in dark conditions and, after 16 h, it was diluted with distilled water to an absorbance of 0.990 ± 0.030 at 734 nm.

In order to evaluate the antioxidant properties of the Calabrian and Chinese Goji extracts, 1 mL of a solution of each tested item (0.3 mg/mL) was added to 1 mL of the final ABTS solution. A control was prepared in the same reaction conditions but using 1 mL of distilled water. The obtained samples were stored in the dark at room temperature and, after 6 min, the absorbance was measured at 734 nm and the ABTS scavenging effect calculated according to Equation (1).

All determinations were repeated in triplicate.

### 2.5. Oxygen Radical Absorbance Capacity (ORAC) Assay

The antioxidant capacities of Goji samples were evaluated using the method described in literature with a slight modification [13]. The test was performed using black-walled 96-well plates, with each well characterized by a final volume of 200 μL. 75 mM Phosphate buffer (pH 7.4) was prepared for making 40 nM Fluorescein and 150 mM AAPH (2,2′-azobis-(2-amidinopropane) dihydrochloride) solutions. The blank well received 25 μL of distilled water, the sample wells received 25 μL of each Goji extract (solutions at different concentrations), while the standard wells were filled with 25 μL of Trolox standard solutions prepared at different concentrations. Then, each well received 150 μL of Fluorescein and the plate was placed into the microplate reader. After 15 min of incubation at 37 °C, 25 μL AAPH were added to each well and fluorescence was recorded every minute for 60 min using an excitation wavelength of 485 nm and an emission wavelength of 520 nm. Radical absorption capacity was calculated using differences in areas under the Fluorescein decay curve (AUC) between the blank and a sample and results were expressed as μmol of trolox equivalents per 100 grams of dried sample (µmol TE/100 g) according to Equation (2):

(2) $$AUC=1+\frac{f_{1}}{f_{0}}+\frac{f_{2}}{f_{0}}+\frac{f_{3}}{f_{0}}+\frac{f_{4}}{f_{0}}+\ldots \frac{f_{60}}{f_{0}}\;$$

### 2.6. β-Carotene–Linoleic Acid Assay

The antioxidant properties of the Calabrian and Chinese Goji extracts were also explored by evaluating the capability to inhibit lipid peroxidation. 

The assay was carried out according to the procedure already reported in [14]. The two Goji extracts were tested at a concentration of 0.15 mg/mL and the antioxidant activity (*A*_0_ × *A*) was expressed as inhibition percentage according to the Equation (3):

(3) $$A_{0}\times A=100\times \left(1-\frac{A_{0}-A_{t}}{{A^{\prime }}_{0}-{A^{\prime }}_{t}}\right)\;$$

where *A*_0_ and *A*′_0_ are the absorbance values recorded at the initial time of the incubation for tested items and control, respectively, and *A_t_* and *A*′*_t_* are the absorbance for tested samples and control after incubation for 60 min. 

All the experiments were carried out in triplicate.

### 2.7. Cholinesterase Inhibitory Assay

Acetylcholinesterase inhibition activity of the Goji extracts was evaluated and the adopted experimental procedure was reported in a previous study [15]. In order to determine the IC_50_ values, the amount of 20 µL of different concentrations of Calabrian and Chinese Goji extracts were added to 40 µL of AChE (2 U/mL in PBS pH 8). This reaction mixture was added to 2 mL of buffer pH 8 (0.1 mM) and allowed to stand for 30 min in an ice bath at 4 °C. In order to remove the interference of the test substances, 20 µL of Physostigmine (0.1 mM) were added in duplicate tubes. Finally, 20 µL of DTNB (0.05 mM in buffer pH 7) and 20 µL of ATCI (0.018 mM in buffer pH 7) were added and the tubes were incubated in a water bath at 37 °C for 20 min. With the aim to evaluate the inhibiting activity of the tested samples, the hydrolysis of acetylthiocholine was monitored and the obtained yellow 5-thio-2-nitrobenzoate was read at 405 nm. The obtained results were expressed as inhibition percentage of the enzyme activity calculated according to Equation (4):

(4) $$Inhibition=\frac{\left(A_{b}-A_{bc}\right)-\left(A_{s}-A_{cs}\right)}{\left(A_{b}-A_{bc}\right)}\times 100\;$$

where *A_b_* and *A_bc_* represent the absorbance of blank and blank positive control, respectively, and *A_s_* and *A_cs_* represent the absorbance of sample and sample positive control, respectively.

Each test was carried out in triplicate and data were expressed as means (SEM).

### 2.8. Nitrite Oxide Scavenging Activity

The scavenging effect of the tested samples on Nitric Oxide was evaluated by the quantification of NO radical generated from Sodium nitroprusside according to the method reported in literature with some modifications [16]. An aliquot of 0.5 mL of each Goji extract (0.9 mg/mL) was added to 2.5 mL of Sodium nitroprusside 5 mM in Phosphate buffer solution (pH 7.4) and the tubes were subsequently subjected to irradiation in the presence of a visible polychromatic light source (25 W tungsten lamp) for 3 h. Then, 0.5 mL of the solution in the test tubes were mixed with 0.5 mL of Griess reagent (1% Sulfanilamide in 5% H_3_PO_4_ and 0.1% Naphthylethylenediamine dihydrochloride). After 5 min, the absorbance of the chromophore formed during the diazotization of nitrite with Sulphanilamide and subsequent coupling with Napthylethylenediamine dihydrochloride was read at 546 nm.

A control sample in the absence of Goji extract was treated under the same experimental conditions, but using 0.5 mL of distilled water.

The Nitrite Oxide scavenging activity was calculated according to the Equation (1) and expressed as inhibition percentage.

All determinations were carried out in triplicate.

### 2.9. Total Carotenoids Content

For this study, 1 g of freeze-dried samples was weighted in a tube and mixed with 1 mL of 5% NaCl aqueous solution. The solution was shaken and sonicated for 15 min, using an ultrasonic bath. Later, 3 mL of Hexane were added to the tube and the mixture obtained was magnetically stirred at room temperature overnight. Then, the mixture was centrifuged at 5000 rpm for 10 min. The supernatant was collected and absorbance was measured at 460 nm using Jasco V-530 UV/Vis spectrometer [17]. The same extraction procedure was followed for both Goji extracts. The amount of total Carotenoids was expressed as milligrams equivalents of Zeaxanthin per gram of extract (mg eq Z/g). A calibration curve was established by using zeaxanthin as the marker compound.

### 2.10. Statistical analysis

All data are presented as mean ± standard deviation (SD) and were carried out using unpaired Student’s *t*-test.*p* values < 0.05 were considered statistically significant.

The inhibitory concentrations of 50% (IC_50_) were obtained by interpolation from linear regression analyses.

## 3. Results

### 3.1. Scavenging of DPPH and ABTS Free Radicals

The antioxidant activity of Calabrian and Chinese Goji extracts was investigated by performing the DPPH and ABTS assays, which represent widely employed in vitro methods for the assessment of the antioxidant properties of natural extracts.

**Figure 1:**
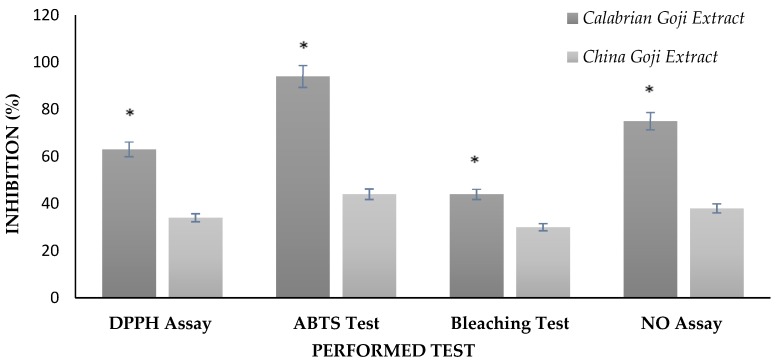
Antioxidant and anti-inflammatory activities of (■) Calabrian and (■) Chinese Goji extracts (0.15 mg/mL) in terms of DPPH, ABTS, lipid peroxidation and NO inhibition expressed as percentage. Values are the mean ± SD (standard deviation). Significance was defined as * *p* < 0.05.

In the present work, the ability of the tested items to reduce DPPH and ABTS radicals was expressed as inhibition percentage and, as it is possible to note in Figure 1, the Calabrian Goji extract exhibited higher antioxidant activities compared to the Chinese one. The tendency of higher inhibition of DPPH and ABTS radicals in Goji berries extract, obtained starting from a Calabrian cultivation, may be attributed to its chemical composition, which may be different from that of the Chinese Goji.

### 3.2. β-Carotene–Linoleic Acid Assay

The β-Carotene–linoleic acid assay, also known as bleaching test, is a measure of the ability of a sample to inhibit lipid peroxidation, which is quantified by the evaluation of β-Carotene discoloration. The obtained results (44.2% ± 1.7% and 30.4% ± 1.1% for Calabrian and Chinese Goji extract, respectively), expressed as inhibition percentage, are shown in Figure 1 and clearly indicate the significant antioxidant properties of the Calabrian Goji extract when compared to the Chinese sample.

### 3.3. Oxygen Radical Absorbance Capacity (ORAC) Assay

**Figure 2:**
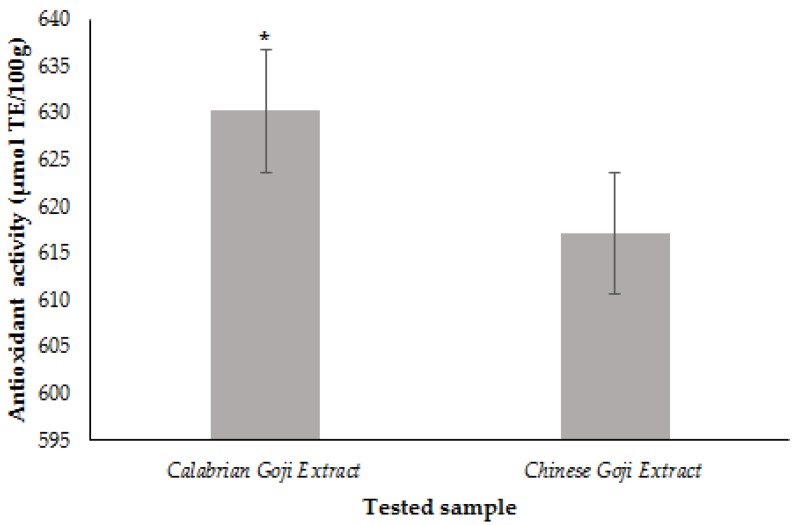
Antioxidant activity (ORAC values) of Calabrian and Chinese Goji extracts. ORAC values are expressed in terms of micromoles of trolox equivalents per 100 g of dry weight. Values are the mean ± SD. Significance was defined as * *p* < 0.05.

Figure 2 shows the antioxidant potential of Calabrian and Chinese Goji extracts evaluated by ORAC assay and expressed as μmol of Trolox equivalents per 100 grams of dried sample (µmol TE/100 g), quantified using a Trolox calibration curve. The Calabrian Goji extract and the Chinese Goji extract showed an antioxidant capacity equal to 630.21 and 617.18 µmol TE/100 g, respectively. In these results, the values obtained for both the tested samples, in term of antioxidant properties, were similar despite the different behavior observed in DPPH and ABTS assays and due to the different nature of the performed methods.

### 3.4. Nitric Oxide Radical Scavenging Assay

In order to investigate the anti-inflammatory properties of Calabrian and Chinese Goji extracts, their Nitric Oxide radical scavenging activity was evaluated in terms of radical reduction and results were expressed as inhibition percentage (75.2%± 1.2% and 38.5% ± 1.6% for Calabrian and Chinese Goji extract, respectively, see Figure 1). The achieved data highlighted the significant effect of Goji fruits cultivated in Calabria on Nitric Oxide, which is a proinflammatory mediator.

### 3.5. Cholinesterase Inhibitory Assay

Calabrian and Chinese Goji extracts were evaluated as AChE inhibiting agents. Both the tested items showed inhibitory effects against the enzyme, with IC_50_ values found to be 10.2 and 14.8 mg/mL for the Calabrian and the Chinese Goji extract, respectively. The obtained results confirmed the relevance of the Calabrian Goji extract as a possible nutraceutical supplementation.

### 3.6. Total Carotenoids as Zeaxanthin Equivalents

The Calabrian and Chinese Goji extracts were analyzed for their content of Carotenoids as Zeaxanthin equivalents, in order to compare these extracts in terms of Zeaxanthin food supplements. Results are shown in Table 1, where the quantitative differences are highlighted among the two samples, probably due to the climatic conditions in which Calabrian Goji was cultivated. The Calabrian Goji has been proved to be a rich source of Carotenoids and, thus, it may be used as Zeaxanthin food supplement in those cases in which the intake of Carotenoids could be useful (e.g., for the improvement of cognitive disorders) [5,7,9].

## 4. Discussion

### 4.1. Antioxidant Properties

In order to verify and compare the antioxidant properties of Calabrian and Chinese Goji extracts, their scavenging ability towards DPPH and ABTS radicals was investigated. The obtained results have evidenced the presence of an important antioxidant activity in both the Goji extracts, but, in the same conditions, Calabrian Goji exhibits a stronger antioxidant ability. This tendency is probably due to the higher concentration of Carotenoids and Zeaxanthin. It is well known, indeed, that Carotenoids are able to prevent damaging oxidation by scavenging free radicals [18]. Furthermore, in another study, the antioxidant activity of Zeaxanthin and Carotenoid fractions was studied and their ability to scavenge hydroxyl and superoxide radical anions was confirmed [19]. The ability of Calabrian and Chinese Goji extracts to inhibit lipid peroxidation was also evaluated. The importance of the assessment of inhibition ability of lipid peroxidation is due to the negative influence towards neurodegenerative disorders [20,21], above all during aging [22]. For this reason, herbal drug supplementation that contains antioxidant compounds could improve the oxidative stress induced by aging and neurodegenerative conditions [23,24].

In order to perform this test, β-Carotene-linoleic acid system was used. The aim of this assay is to observe the β-Carotene bleaching, an event that occurs only in the absence of an antioxidant. After the oxidation of Linoleic acid, free radicals are generated and themselves attack β-Carotene molecules, which lose their conjugation and undergo discoloration. In the presence of an antioxidant, the structure and the orange color of β-Carotene are maintained [15]. The results confirmed the higher antioxidant activity of the Calabrian Goji compared to the Chinese Goji.

Furthermore, ORAC assay was employed to evaluate the total antioxidant capacity. This method measures the ability of antioxidant compounds, present in the tested samples, to inhibit the decline of R-PE fluorescence that is induced by a peroxyl radical generator, such as AAPH [25]. In this test, the obtained results showed little difference between Calabrian and Chinese Goji extract. In both Goji samples, a similar total antioxidant activity was observed.

### 4.2. Cholinesterase Inhibitory Activity

In a previous study, the pathogenesis of several neurodegenerative disorders (ND) was studied and the obtained results have shown that the oxidative stress and injured cholinergic neurotransmission play an important role in the development of these disorders [26]. In order to treat ND, several drugs that are able to improve brain cholinergic activity by inhibiting Acetylcholinesterase enzyme (AChE) were used [27]. The inhibition of this enzyme could be a possible approach to delay neurodegenerative disease progression, but the use of this kind of therapeutic agents may cause several side effects. For all these reasons, several natural products which can be used as AChE inhibitors, but with fewer side effects, have replaced some of these drugs [28]. In order to verify their ability to inhibit AChE and, thus, to improve these neurodegenerative disorders, Calabrian and Chinese Goji extracts were tested. Also in this test, Calabrian Goji extract exhibited the strongest inhibiting activity, confirming that its levels of Carotenoids and Zeaxanthin are probably related to its ability to inhibit AChE.

### 4.3. Nitric Oxide Radical Scavenging Ability

In the aim to evaluate the anti-inflammatory activity of Calabrian and Chinese Goji extracts, their reactivity towards Nitric Oxide (NO^.^) was assessed; if Nitric Oxide is present in high concentrations, indeed, it can be considered a proinflammatory mediator [29]. NO is able to react with superoxide and thus produce peroxynitrite anion, a potential strong oxidant that triggers oxidative damage [30]. For all of these reasons, the ability to scavenge Nitric Oxide is used to investigate in vitro the anti-inflammatory properties of several natural samples. In a previous work, loss of memory (Alzheimer’s disease) and other cognitive disorders were studied and the cause of their development was found to be inflammatory damage [31]. This research was confirmed by another study that have found high levels of inflammatory markers in the central nervous system of individuals with cognitive (Alzheimer’s disease) and neurodegenerative disorders [32]. A possible remedy to improve neurologic and cognitive development is to introduce antioxidant and anti-inflammatory agents through the diet; an example of these is Zeaxanthin [33]. In this study, Calabrian Goji extract was found to have good anti-inflammatory activity and this result could be attributed to its high levels of Carotenoids, particularly in terms of Zeaxanthin.

### 4.4. Carotenoids Quantification in Zeaxanthin Equivalents

Most of the biological activities of Goji fruits are attributed to their Carotenoids content. Carotenoids, indeed, are the second biological constituent of Goji and, among them, the xanthophyll Zeaxanthin, present in the form of Dipalmitin Zeaxanthin, is the most representative [34]. Therefore, Goji could be considered a valid natural source of Dipalmitin Zeaxanthin [34]. In order to investigate the differences in Carotenoids content between two Goji fruits, grown in two different climatic conditions, the quantification of Carotenoids in terms of Zeaxanthin was carried out and the obtained results were compared. It is well known that the intensity of temperature and the sun exposure of fruits influence their chemical composition and, in particular, their Carotenoids content. For these reasons, summer fruits like Goji accumulate more Carotenoids than fall fruits and present high levels of Zeaxanthin dipalmitate, the major pigment and colorant matter of the fruit [35]. The results of this study confirmed that the sun exposure and the Mediterranean climatic conditions of the Sibari Plain increase the levels of Carotenoids and Zeaxanthin in Goji fruit. The extract obtained from the Calabrian Goji, indeed, has higher levels Carotenoids in terms of Zeaxanthin in comparison to the extract obtained from the Chinese Goji. All these findings suggest that Calabrian Goji extract, due to its biological properties and high Carotenoids content, including Zeaxanthin, might be used as a food supplement for the treatment of cognitive disorders, mental impairments and other neurodegenerative diseases. In fact, carotenoids are reported to be optimal supplementation for the improvement of such conditions [5,7,9].

## 5. Conclusions

In the present research study, the biological and functional properties of Goji extract, obtained from plants cultivated in Calabria, were evaluated and compared to those of an extract prepared in the same conditions but using Chinese Goji fruits. In this context, the antioxidant properties were investigated by performing DPPH, ABTS, ORAC and β-Carotene–linoleic acid assays; furthermore, the ability to inhibit Acetylcholinesterase activity and to scavenge Nitric Oxide radicals was also explored. Finally, the total Carotenoids content was quantified and expressed in terms of Zeaxanthin. The obtained results highlighted the good antioxidant, anti-inflammatory and anticholinesterase in vitro properties of the Calabrian Goji extract, which was also found to be a rich source of Carotenoids, compared to the Chinese Goji extract. These findings suggest the potential use of Calabrian Goji extract as a food supplement for cognitive and mental disorders, due to its beneficial effects on human health.

## Figures and Tables

**Table 1:** Total Carotenoids expressed as milligram equivalents of Zeaxanthin per gram of extract (mg eq Z/g) in Calabrian and Chinese Goji extracts.

Samples	Total Carotenoids Content (mg eq Z/g)
Calabrian Goji extract	7.8 ± 0.1
Chinese Goji extract	5.2 ± 0.3 ^1^

^1^ Values are expressed as the mean ± SD of at least three independent experiments.
